# A Case of Severe Hyponatremia in a Patient With Primary Adrenal Insufficiency

**DOI:** 10.7759/cureus.17946

**Published:** 2021-09-13

**Authors:** Sandhya Shanthosh Kumar, Vignesh Krishnan Nagesh, Jake Hunter, Ibrahim Sange

**Affiliations:** 1 Internal Medicine, Rajarshee Chhatrapati Shahu Maharaj Government Medical College, Kolhapur, IND; 2 Internal Medicine, Stony Brook Southampton Hospital, New York, USA; 3 Internal Medicine, Government Medical College Thiruvananthapuram, Thiruvananthapuram, IND; 4 Medicine, K. J. Somaiya Medical College, Mumbai, IND

**Keywords:** severe hyponatremia, primary adrenal insufficiency, siadh, glucocorticoid, mineralocorticoid, hypotonic hyponatremia, renin, aldosterone, tsh, desmopressin

## Abstract

Primary adrenal insufficiency leads to the decreased production of cortisol and aldosterone. Patients develop an electrolyte imbalance, which can be severe and life-threatening. It is more common in women, usually between the second and fourth decades.

We present the case of a 58-year-old female who was admitted due to laboratory findings of severe hyponatremia. Further workup revealed the underlying cause to be primary adrenal insufficiency. Our case report highlights that profound hyponatremia can present with minimal non-specific symptoms and the absence of any neurological manifestations. Correction of hyponatremia, treatment with hydrocortisone and fludrocortisone, and water restriction in the management of this patient resulted in full clinical recovery.

## Introduction

Primary adrenal insufficiency leads to decreased production of glucocorticoids and mineralocorticoids by the adrenal cortex. It can be potentially life-threatening and is estimated to have a current prevalence of about 100-140 cases per million, thereby making it rare. Although 85% of cases of primary adrenal insufficiency are due to autoimmune adrenalitis, other causes, such as infections, tuberculosis, and adrenal hemorrhage, should also be considered [1-2].

Clinical presentation usually results from associated electrolyte disturbances, commonly hyponatremia, and hyperkalemia [3], and patients usually present with diverse non-specific symptoms. These include nausea, vomiting, poor appetite, unexplained fatigue, chronic abdominal pain, weight loss, confusion, dizziness, hyperpigmentation, salt cravings, and orthostatic hypotension [4-5]. The severity of symptoms depends upon the extent of involvement of the adrenal gland and the associated hormonal deficit [3,5].

This disease usually follows a slow and insidious course, leading to worsening of symptoms over time. This makes early detection challenging and often results in delayed diagnosis [6]. Hyponatremia is associated with decreased glucocorticoid activity, which can be further aggravated by mineralocorticoid deficiency, and results in high morbidity and mortality [7]. A thorough comprehensive metabolic panel along with an estimation of adrenal hormones is necessary to arrive at a definitive diagnosis. Exogenous replacement of glucocorticoids, as well as mineralocorticoids, is generally required to treat these patients [8-9].

## Case presentation

A 58-year-old female presented to the emergency department with nausea, vomiting, and fatigue for three to four days. She also experienced lightheadedness associated with anxiety, palpitations, as well as blurry vision occasionally. Her last routine health checkup was five years ago, and her past medical history was unremarkable. The patient was feeling unwell for at least one month, during which she experienced a lack of appetite, early satiety, malaise, and unintentional weight loss. The patient also recalled developing an unusually strong craving for pickles for several weeks before her symptom onset. She also reported occasional abdominal pain as well as leg cramps. The patient attributed the origin of her symptoms to the stressful period in her life following the demise of her father. However, she denied any complaints of dizziness on standing, hypoglycemic episodes, tanning of skin or mucous membranes.

On admission, her blood pressure was 103/63 mmHg, and her heart rate was 79 beats/min. Orthostasis was negative. The patient was ill-appearing but had no associated neurological deficits. Physical examination was otherwise unremarkable and showed no signs of dehydration. Blood chemistry revealed significantly low serum sodium of 109 mmol/L, chloride of 77 mmol/L, potassium of 4.8 mmol/L, total bilirubin of 1.4 mg/dl, and glucose of 117 mg/dl. Complete blood count showed hemoglobin of 14.1 g/dL, hematocrit of 38.6%, RBC count of 4.79 10^3/µL, and WBC count of 8.81 10^3/µL. Further tests revealed decreased plasma osmolality (230 mOsm/kg), elevated urine osmolality (705 mOsm/kg), elevated urine sodium (131 mmol/L), and normal renal function. Her findings were consistent with euvolemic hypotonic hyponatremia. Additional tests to evaluate adrenal function were done, which revealed significantly elevated adrenocorticotropic hormone (ACTH) level (1286 pg/dl), low aldosterone level (1 ng/dl), and elevated plasma renin level (60.34 ng/ml/hr). The morning cortisol level for this patient was in the low-normal range (6.2 mcg/dl). An ACTH stimulation test was then done, which failed to raise cortisol levels in this patient. Table 1 summarizes these significant findings, which ultimately led to a definitive diagnosis of primary adrenal insufficiency in this patient. The rest of the biochemical analysis, including thyroid-stimulating hormone (TSH), calcium, phosphorus, total protein albumin, lipid panel, and globulin, was within normal range. All other relevant investigations, including electrocardiogram, chest X-ray, computed tomography (CT) brain (without contrast), CT angiography head and neck (with contrast), CT chest/abdomen/pelvis, and magnetic resonance imaging (MRI) brain and pituitary, were done to rule out any secondary causes of hyponatremia were negative.

**Table 1:** Laboratory results of the patient ACTH: adrenocorticotropic hormone

Labs	Patient's Results	Reference Range
Plasma sodium	109	135-145 mmol/l
Potassium	4.8	3.5-5 mmol/l
Cortisol	6.2	5-23 mcg/dl
ACTH	1286	9-52 pg/dl
Aldosterone	1	2-9 ng/dl
Plasma renin	60.34	0.6-4.3 ng/ml/hr
Plasma osmolality	230	275-295 mOsm/Kg
Urine osmolality	705	50-1200 mOsm/Kg

The patient was treated initially with hypertonic saline along with a dose of desmopressin for the correction of severe hyponatremia. Once the patient’s sodium level was gradually corrected to above 120 mmol/l, hypertonic saline was discontinued. The patient also received treatment with hydrocortisone and fludrocortisone, along with fluid restriction of one liter of normal saline per day. The normalization of the serum sodium levels over the next few days resulted in the resolution of symptoms and direct improvement of the patient's clinical status. The patient was discharged on fludrocortisone, hydrocortisone, and salt tablets.

## Discussion

Adrenal failure results in an initial decrease in cortisol levels followed by a subsequent reduction in aldosterone levels depending upon the extent of involvement of the adrenal gland. This leads to a subsequent elevation in ACTH and melanocyte-stimulating hormone (MSH) levels due to the lack of negative feedback inhibition [10].

Hyponatremia is a common electrolyte disturbance that is seen in the setting of adrenal insufficiency, which occurs due to an increase in the antidiuretic hormone (ADH) levels subsequent to cortisol deficiency resulting in the retention of free water [7]. Cortisol deficiency also leads to an increase in cortisol-releasing hormone (CRH) production from the hypothalamus, which is an ADH secretagogue. Cortisol has a negative feedback on CRH and ACTH [11], which is lacking in patients with adrenal insufficiency. Cortisol also causes direct suppression of ADH secretion. Therefore, decreased plasma levels of cortisol result in increased ADH levels, resulting in free water retention and hyponatremia [7]. However, clinicians should be vigilant to rule out any other underlying causes of hyponatremia in a patient, as they can easily be overlooked.

In our case, initial clinical examination and laboratory findings indicated the presence of normovolemic hypotonic hyponatremia. Some of our differential diagnoses under consideration included syndrome of inappropriate anti-diuretic hormone (SIADH), adrenal insufficiency, hypothyroidism, diuretic abuse, or glucocorticoid deficiency. In this patient, the blood workup revealed significantly elevated ACTH levels (1286 pg/dl), low aldosterone levels (1 ng/dl), and elevated plasma renin levels (60.34 ng/ml/hr) which indicated both glucocorticoid and mineralocorticoid deficiency. The morning cortisol levels for this patient were in the low-normal range (6.2 mcg/dl). An ACTH stimulation test was then done, which failed to raise cortisol levels in this patient. Furthermore, the low aldosterone level, along with the lack of cortisol-mediated negative feedback on ADH resulted in severe hyponatremia (109 mmol/l) in this patient over a period of time. The chronicity of adrenal insufficiency, in this patient, resulted in a less severe clinical presentation as compared to acute cases of severe hyponatremia. In conclusion, the low aldosterone levels and low-normal cortisol levels, lack of adrenal response to the ACTH stimulation test, as well as the markedly elevated ACTH levels and renin levels, together led to a definitive diagnosis of primary adrenal insufficiency in this patient.

Primary adrenal insufficiency also manifests as hyperpigmentation (due to increased MSH levels) and hyperkalemia (due to low aldosterone) as part of its presentation [12]. However, these findings were missing in our patient. Further workup in the patient also revealed decreased plasma osmolality (230 mOsm/kg), elevated urine osmolality (705 mOsm/kg), and elevated urine sodium (131 mmol/L). The patient on evaluation was found to be euvolemic. The decreased serum osmolality, increased urine osmolality, increased urine sodium, as well as euvolemia, were consistent with SIADH, possibly secondary to adrenal insufficiency in this patient. This patient had normal thyroid-stimulating hormone levels so hypothyroidism was ruled out as a cause of hyponatremia.

The serum creatinine, blood urea nitrogen levels, and glomerular filtration rate were normal for this patient, and there were no signs of volume depletion or overload to suggest renal insufficiency. The patient also had no history of any medical conditions requiring the use of diuretics. Also, her blood work ruled out any causes for pseudohyponatremia (such as hyperlipidemia, hyperproteinemia, and marked hyperglycemia).

Treatment for primary adrenal insufficiency focuses on replenishment of the hormone deficit. Glucocorticoids are replaced through the administration of hydrocortisone and occasionally oral prednisone. Mineralocorticoids are replaced through the administration of fludrocortisone to restore the electrolyte balance [2,5]. This patient was treated with hydrocortisone and fludrocortisone and was kept on a fluid restriction of one liter of normal saline per day. Additionally, in this patient, the administration of hypertonic saline and desmopressin was necessary initially due to the severity of hyponatremia. This patient showed complete normalization of hyponatremia and resolution of symptoms following treatment and was discharged on medications with instruction to follow up regularly. Patients with this condition usually require lifelong maintenance with medications and follow-up.

## Conclusions

Hyponatremia is commonly encountered in clinical practice. However, our case report highlights that chronic severe hyponatremia can occur in association with primary adrenal insufficiency and that profound hyponatremia of gradual onset, with a sodium level as low as 109 mmol/L, could have absolutely no neurological symptoms due to the remarkable adaptation of the brain because of its chronic, insidious course. Severe hyponatremia is often multifactorial, as in this case, due to primary adrenal insufficiency and SIADH (secondary to adrenal insufficiency). Therefore, a thorough evaluation must be done to uncover all the predisposing conditions and determine the underlying cause of hyponatremia.
